# Multigeneration Cross-Contamination of Mail with *Bacillus anthracis* Spores

**DOI:** 10.1371/journal.pone.0152225

**Published:** 2016-04-28

**Authors:** Jason Edmonds, H. D. Alan Lindquist, Jonathan Sabol, Kenneth Martinez, Sean Shadomy, Tyler Cymet, Peter Emanuel

**Affiliations:** 1 Edgewood Chemical Biological Center, United States Army, Department of Defense, 5183 Blackhawk Road, Aberdeen Proving Ground, MD, 21010, United States of America; 2 National Homeland Security Research Center, Office of Research and Development, U.S. Environmental Protection Agency, 26 West Martin Luther King Drive, Cincinnati, OH, 45268, United States of America; 3 HWC Inc., 1100 New York Avenue, NW, Washington, DC, 20005, United States of America; 4 National Center for Emerging and Zoonotic Infectious Diseases, United States Centers for Disease Control and Prevention, 1600 Clifton Road, MS A-30, Atlanta, GA, 30333, United States of America; 5 Associate Vice President for Medical Education, American Association of Colleges of Osteopathic Medicine, Chevy Chase, Maryland, 20815, United States of America; ContraFect Corporation, UNITED STATES

## Abstract

The release of biological agents, including those which could be used in biowarfare or bioterrorism in large urban areas, has been a concern for governments for nearly three decades. Previous incidents from Sverdlosk and the postal anthrax attack of 2001 have raised questions on the mechanism of spread of *Bacillus anthracis* spores as an aerosol or contaminant. Prior studies have demonstrated that *Bacillus atrophaeus* is easily transferred through simulated mail handing, but no reports have demonstrated this ability with *Bacillus anthracis* spores, which have morphological differences that may affect adhesion properties between spore and formite. In this study, equipment developed to simulate interactions across three generations of envelopes subjected to tumbling and mixing was used to evaluate the potential for cross-contamination of *B*. *anthracis* spores in simulated mail handling. In these experiments, we found that the potential for cross-contamination through letter tumbling from one generation to the next varied between generations while the presence of a fluidizer had no statistical impact on the transfer of material. Likewise, the presence or absence of a fluidizer had no statistically significant impact on cross-contamination levels or reaerosolization from letter opening.

## Introduction

The unintentional release of *Bacillus anthracis* (*Ba*) in Sverdlovsk, Russia in April of 1979 from a suspected biological warfare agent (BWA) production facility resulted in the death of at least 68 individuals in the surrounding population [1]. Although unintentional, this was the first documented example of the hazard posed with the release of a BWA in a civilian population. In September and October of 2001, letters containing *Ba* spores were intentionally placed into and distributed through the United States Postal Service (USPS) in pre-stamped envelopes [2]. These mailings of spores resulted in contamination of 35 commercial mail rooms as well as processing and distribution centers in Hamilton, New Jersey, Washington D.C., New York City, and Wallingford, Connecticut [2]. Additionally, postal facilities along the path transited by contaminated letters mailed to a targeted media company in Florida became contaminated.

Subsequent to this bioterrorist attack, now known as the Amerithrax event, 22 individuals, including postal workers, persons who received or handled the contaminated letters, and persons exposed to environments contaminated by the letters, developed either inhalation or cutaneous anthrax; five of these individuals died from inhalation anthrax [2–7]. An additional 43 individuals tested positive for exposure to *Ba* spores by nasal swab culture as part of the public health response to the attacks, including 38 individuals working at the Hart Senate Office Building and 5 individuals working at the America Media Inc. building in Boca Raton, Florida. These persons were provided oral post-exposure antimicrobial prophylaxis, with or without anthrax vaccine adsorbed (AVA), and did not develop anthrax [2, 8]. Subsequent studies have further investigated the relationships between infection or evidence of exposure and physical presence in areas where contamination was known to be present, or where other known exposures occurred [8–10]. These additional studies suggest that additional persons may have been exposed beyond those identified during the Amerithrax event and response and the anxiety created by uncertainty increased the effect of these attacks [11–12].

With an uncertain and unpredictable pattern of the spread of contamination, developing a system to identify potentially affected buildings and surrounding areas in order to provide postexposure prophylaxis (PEP) to populations in those areas and conduct decontamination is difficult [13]. Even in light of recent investigations into this phenomenon, the mechanics of the transmission of anthrax spores and resulting exposure and infection are poorly understood and likely a complex process. This is best exemplified by the cases of two individuals who developed inhalational anthrax during the 2001 Amerithrax event, an elderly woman in Connecticut and a hospital supply worker in New York City, where subsequent investigations failed to detect *Ba* spores on their mail, or in their homes, vehicles, or locations they frequently visited [6–7, 14–15]. However, positive samples were found in a mail office a mile from the home of the Connecticut woman [6, 16–17].

Without evidence of the presence of *Ba* spores in locations frequented by the inhalation anthrax cases from Connecticut and New York City it is impossible to demonstrate the source of their infections. Hypothetically their infections may have resulted from exposure to mail contaminated by passing through the same sorting equipment around the same time as recovered spore-containing letters that were sent to Senators Leahy and Daschle or others were processed, or cross-contaminated by such envelopes [18]. Due to the lack of additional documented cases in these areas or supporting environmental sampling data, there is no way to confirm this as a potential route of exposure. A previous hypothesis suggested that these individuals may have become infected through inhalation exposure to spores released from spore-containing envelopes that they, or someone in proximity to them, tore open and then discarded [19]. The delay between exposure and disease may have led to the contaminated mail being discarded. In this instance, the possibility of the fomite responsible for transmission having been discarded and removed, combined with the incomplete data on the limits of detection for environmental sampling or a low concentration of spores remaining on environmental surfaces, may have contributed to the source of exposure remaining undetected [20–21].

The exposure of these two inhalation anthrax cases may also have resulted from handling secondary- or tertiary-contaminated mail. A previous study investigating the reaerosolization of spores from contaminated mail demonstrated that a significant number of spores can become airborne through the process of opening cross-contaminated mail [19]. This is of particular concern because it demonstrates the potential for exposure without a requirement to come into physical contact with contaminated surfaces.

Re-aerosolization is a poorly studied phenomenon dependent upon a number of known forces such as van der Waals, electrostatic, adhesion, and surface tension of adsorbed liquid, as well as the potential for additional unknown forces which dictate adhesion and release of particles from substrates [22]. Predicting the adhesion of particles containing biological organisms is more difficult due to the presence of residual media, preparation protocols, organism structures such as exosporium surface proteins, and other physiological differences which exist between not only species, but even between strains within a single species and between methods of preparation. In a previous study, a device was designed to expose uncontaminated envelopes to envelopes bearing a powdered preparation of *Bacillus atrophaeus* var. *globigii* (BG) spores, and then to expose additional uncontaminated envelopes to envelopes that had been exposed to the initial powder-bearing envelope [19]. This device was designed and demonstrated to transfer spore-containing particles between envelopes in a consistent, reproducible manner. In those experiments, the surrogate BG used has a crystalline protein on the outer surface of the spore that is lacking in *Ba*. Additionally, *Ba* contains hair-like fibers on the exosporium membrane, lacking on BG [23–24]. As a consequence the BG surrogate studies may have produced results not directly comparable to *Ba*. It is unclear if these features could play a role in fomite affinity and whether data using BG is an adequate simulant for the human pathogen *Ba*.

## Materials and Methods

### Spore Preparation

Two preparations of the *Bacillus anthracis* Sterne vaccine strain (BAS), lot #28Apr2010WSJ, were supplied by Dugway Proving Grounds (U.S. Army). In both preparations, spore-containing particles were milled to a median aerodynamic diameter of 1.42 μm. The median mass diameter was 6.14 μm, with a mean mass diameter of 10.45 μm. Measured spore concentration in the preparation was 5.1 x 10^9^ CFUs g^-1^. One preparation of spores was left unaltered while the second preparation of spores was mixed with 5% Aerosil R976S (Nippon Aerosil Co., Ltd.).

### Tumbler

The tumbler was developed by the Advanced Design and Manufacturing Team at Aberdeen Proving Ground and was designed to tumble a group of letters at a standard speed of 3 revolutions per minute. The tumbler consisted of an outer sealed container, and an inner stainless steel insert with fins designed to facilitate mixing, and a mechanism for rotating the outer sealed container. A detailed description and figures of the tumbler has been previously described in Edmonds et. al 2010 [19].

### Loading of the payload letter

One standard sized 10.48 cm X 24.13 cm (No. 10 business size) envelope was stuffed with one 21.59 cm X 27.94 cm (letter size) tri-folded piece of plain white printer paper containing one gram of dry BAS milled spores with or without 5% fluidizer as dictated by the experiment. The envelope was then sealed using a moistened cotton swab (Puritan; Fisher Scientific, Suwanee, GA; catalog no. 14-959-102).

### Surrogate Transfer Study: Primary, Secondary, and Tertiary Contamination

#### Primary Contamination

**Fig 1:**
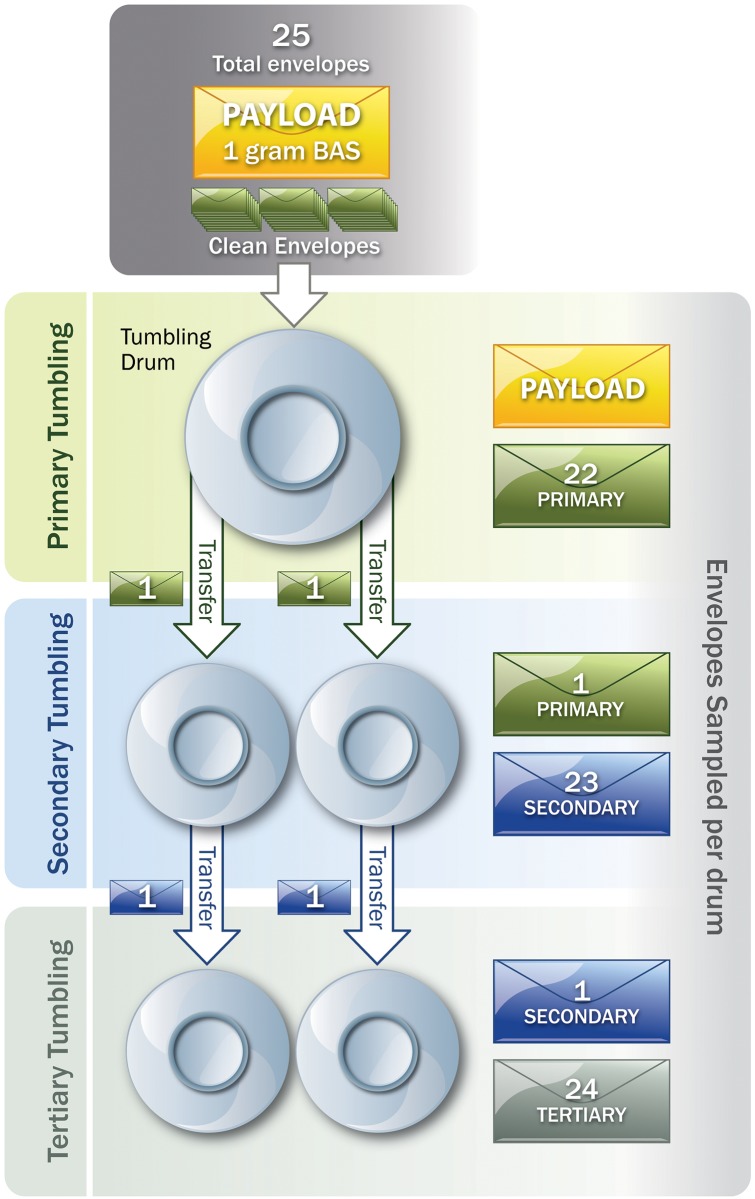
Flow chart of letter tumbling process. Image reproduced from Edmonds et al 2010 [19].

One 19 L bucket with a 30.5 cm gamma seal lid (Pleasant Hill Grain, Aurora, NE) was placed inside the tumbler device. With the lid off, a sterile stainless steel insert [19] was placed inside the bucket with the set screws screwed down tightly. Twenty-four 10.48 cm X 24.13 cm (No. 10 business size) envelopes were each stuffed with one sheet of 21.59 cm X 27.94 cm (letter size) tri-folded white printer paper and sealed as described above and numbered sequentially with pencil in the center and corners. The stuffed and sealed envelopes were placed inside the stainless steel insert. One payload letter was placed inside the stainless steel insert, on top of the 24 sealed envelopes. Five 47-mm glass fiber filters (Pall Life Sciences, VWR, West Chester, PA) were then placed on top of the payload letter. The lid was secured tightly and the bucket was tumbled for one hour at 3 rpm. After one hour, the tumbler was turned off and envelopes remained inside the tumbler for 30 minutes. The envelopes were then removed individually with their order and orientation recorded. The first two envelopes, excluding the top envelope or the envelope located immediately above and below the payload envelope, were selected and set aside for secondary contamination (Fig 1). The remaining envelopes and glass fiber filters were placed into individual stomacher bags (Seward BA6141, West Sussex, UK) containing 35 mL of sterile deionized water and sealed. Each sample was mixed by a stomacher (Seward Circulator 400, West Sussex, UK) for two minutes at 260 rpm. Using a sterile pipet, the remaining liquid not absorbed by the envelope was removed, volume recorded, and the liquid was placed into a 50 cc snap-cap conical tube (NUNC, Thermo Fisher Scientific, Rochester, NY). Each sample was then diluted to extinction with 50 uL of deionized H_2_O plated on tryptic soy agar (TSA, Becton Dickenson, Sparks, MD) in triplicate using a spiral plater (Spiral Biotech, Norwood, MA). Colonies were counted and CFU counts were stored electronically by Q-count (Spiral Biotech, Norwood, MA) with the final spore number calculated (Total CFU) for both controls and samples.

#### Secondary and Tertiary Contamination

A new 19 L bucket with gamma seal lid was placed inside the tumbler device, along with a sterile stainless steel insert. One envelope set aside from the primary or secondary contamination was placed on top of 24 stuffed and sealed envelopes labeled sequentially as described above, identical to the procedure followed in the primary contamination experiment (Fig 1). Five additional glass fiber filters were placed on top of the contaminated letter. The gamma seal lid was secured tightly and the envelopes were tumbled for one hour at 3 rpm. The envelopes were again allowed to rest for 30 minutes before processing. Two envelopes were set aside from the secondary contamination to use as a seed for the tertiary contamination using the same criteria as in the primary contamination. All remaining envelopes from all runs were recorded and processed in the same manner as in the primary contamination. Two runs of each generation (primary, secondary, and tertiary) were performed in this manner.

### Hazard of Operation Study: Opening Contaminated Letters (Simulant)

Twenty-four primarily contaminated envelopes were generated through tumbling in the manner described. Fifteen envelopes, excluding the top envelope and the envelopes located immediately above and below the payload envelope, were selected in the order that they came out of the tumbling drum and set aside for aerosol release testing. Five envelopes were used for control enumeration, and five envelopes were used for each of the two tearing test protocols for a total of 15 envelopes used per tumbling event. The payload letter and nine of the cross-contaminated letters were discarded. The air sampling protocol involved tearing, with a finger, the width of the envelope from the top corner where the postage stamp would be located, to the bottom corner of that same side. One envelope at a time was placed inside a 180 L glove box containing two air filter devices with 47-mm glass fiber filters. The bottom surface of the glove box was covered by aluminum foil to reduce the likelihood of cross-contamination between samples. A mass flow meter (Alicat Scientific, Tucson, AZ) was then connected to each air filter device and pump to regulate the flow of air through the glass fiber filters. Each mass flow meter was set to regulate the vacuum pumps to pull air at a rate of 20 L/min with HEPA filtered make-up air brought into the system to replace the sampled air. All pumps were turned on simultaneously 5 seconds prior to sampling of the letter. The air pumps remained on and sampled the air for 18 minutes (four air exchanges). After air sampling, both of the glass fiber filters were removed from the air filter device and placed into the same stomacher bag containing 35 mL of sterile deionized water and sealed. Each sample was then placed inside a stomacher for two minutes at 260 rpm and further processed as previously described. This procedure was repeated for each of the five envelopes for the two tearing protocols. The glove box interior surfaces were cleaned with bleach, deionized water and drying cloth after each envelope sampling. The aluminum foil was discarded. The five control envelopes set aside for enumeration were placed in separate stomacher bags containing 35 mL of sterile deionized water and sealed and processed as earlier described. This process was repeated for both BAS with and without 5% fluidizer.

### Statistical Analysis of Envelope Randomization

The degree of randomness of the envelopes was analyzed concurrently with the addition of biological material during the course of the experiment. Confirmation of random tumbling events was confirmed by statistical analysis as reported in Edmonds *et al*. 2010 [19]. Data upon which the degree of randomness could be assessed were acquired based on the numerical order in which the envelopes were removed from the tumbler (after tumbling), as well as analysis of the orientation of the envelopes when removed (flap facing up or down, to the left or right, forward or back). The data were then analyzed for randomization using the Wald-Wolfowitz runs test and the Mann-Kendall test. These two tests were based on the length of a run of consecutive events, such as numerical order and flap orientation, as well as the number of defined runs. A statistically random experiment was accepted at a 95% confidence level (Edmonds *et al*. 2010). The same protocol and equipment under the same environmental controls was used for the work presented here and thus, we did not repeat the statistical analysis for randomization with the rigor documented in Edmonds *et al*. 2010.

## Results

### Transfer of spores to subsequent generations of envelopes

Primary tumbling of a letter loaded with one gram of BAS spores consisting of 5 x 10^9^ CFUs g^-1^ produced a mean cross-contamination of 1.4 X 10^5^ CFUs per envelope and reduced the number of CFUs retained within and on the payload envelope to 1.9 X 10^8^ CFUs. After tumbling took place and the lid from the apparatus was removed, it was observed that approximately 100mg– 200mg of the spore powder preparation had settled to the bottom of the bucket after completion of each run of the experiment. However, the powder was never collected and weighed, and only visual approximations were recorded. After the secondary tumbling, the transfer envelopes from the primary tumbling, which were the source of contamination, were found to have retained 1.4 X 10^5^ CFUs. These letters produced an average cross-contamination of 2.8 X 10^3^ CFUs per envelope. The envelopes which were cross-contaminated in the tertiary tumbling consisted of 2.1 X 10^3^ CFUs on average. The transfer envelopes from the second tumbling that provided the source of contamination retained 3.5 X 10^3^ CFUs (Table 1).

**Table 1:** CFU recovery following three generations of *Bacillus anthracis* Sterne mail tumbling.

	Primary Tumbling	Secondary Tumbling	Tertiary Tumbling
	CFUs (CV*%)	CFUs (CV%)	CFUs (CV%)
BAS Load Envelope	1.90E+08 (78%) n = 3	1.40E+05 (131%) n = 6	3.50E+03 (137%) n = 6
Paper Stuffed envelopes	1.4E+05 (76%) n = 66	2.8E+03 (194%) n = 138	2.1E+03 (183%) n = 144
BAS Fluidized Load Envelope	1.20E+08 (156%) n = 3	3.20E+04 (139%) n = 6	1.40E+03 (41%) n = 6
Paper Stuffed envelopes	3.5E+05 (76%) n = 66	5.4E+03 (146%) n = 138	1.1E+03 (221%) n = 144

*Coefficient of Variation

Primary tumbling of a letter loaded with one gram of BAS spores with 5% fluidizer consisting of 5 x 10^9^ CFUs g^-1^ produced a mean cross-contamination of 3.5 X 10^5^ CFUs per envelope and reduced the number of CFUs contained within and on the payload envelope to 1.2 X 10^8^ CFUs. After the secondary tumbling, the transfer envelopes which were the source of contamination were found to retain 3.2 X 10^4^ CFUs. These letters produced an average cross-contamination of 5.4 X 10^3^ CFUs per envelope. The envelopes which were cross-contaminated in the tertiary tumbling had been contaminated with 1.1 X 10^3^ CFUs on average. The letters providing the source of contamination retained 1.4 X 10^3^ CFUs (Table 1).

### Re-aerosolization of spores from opened mail

Letters contaminated from the primary tumbling with payload letters containing either BAS spores or BAS spores + 5% fluidizer were opened using a finger. When letters cross-contaminated with BAS spores were opened, 1.9 X 10^3^ aerosolized CFUs were recovered from the air leaving 1.7% of the spores bound to the envelopes. When letters cross-contaminated with BAS spores + 5% fluidizer were opened, 6.1 X 10^3^ aerosolized CFUs were recovered from the air resulting in 1.4% of the initial contamination remaining on the envelopes. There was no statistically significant difference in reaerosolization percentage between BAS and BAS with 5% fluidizer.

## Discussion

Despite attempts to perform a direct comparison between the previous BG studies and the BAS data presented here, inherent differences in the spore preps likely exist which could have contributed to some of the differences in cross-contamination and aerosolization. Although all spore preps were obtained from the same production facility around the same time, it is highly unlikely that the spore preps were identical in production regardless of how closely protocols were followed. Likewise, the starting concentration of the BAS was two logs less than the BG. Despite the difference in publication years between this manuscript and the 2010 manuscript originally describing the protocol and equipment used, the experimental data were generated in a much closer timeframe by the same personnel, utilizing the same protocols, equipment and under the same environmental controls, controlling as best as possible for inter-laboratory and inter-operator variability. However, because the studies were not run in parallel, starting material varied significantly, and two separate spore preps were used, it is possible that some differences in cross contamination and reaerosolization could be due to unknown variations in procedure making a direct comparison inappropriate.

Surrogates are routinely used for experimental analysis for research involving pathogenic biological organisms. The higher degree of safety, better access to material, and less stringent requirements for storage, tracking, and use of surrogates in comparison to pathogenic microorganisms offers a cost and time benefit over the use of pathogenic BWAs. Regardless of the similarity in phenotype or genotype, often times the phenomenology between two separate species, strains, or methods of preparation vary such that no or limited correlations between pathogen and surrogate can be made. In the previous analysis of the spread of contamination through mail by a tumbling process, *Ba* spores were not used, but instead BG was used in its place [19]. In that study it was found that there was approximately a 2 log decay in the number of spores found on the newly cross-contaminated envelopes compared to the seed envelope, between generations of mail tumbling [19]. In an attempt to try to better understand and characterize the transfer of material between envelopes during transit and processing in the USPS which took place during the 2001 anthrax attack, the present study used *Bacillus anthracis* Sterne spores. Additionally, spore preparations with and without a fluidizing agent were used to address concerns about differences in the behavior of material prepared under different conditions.

**Fig 2:**
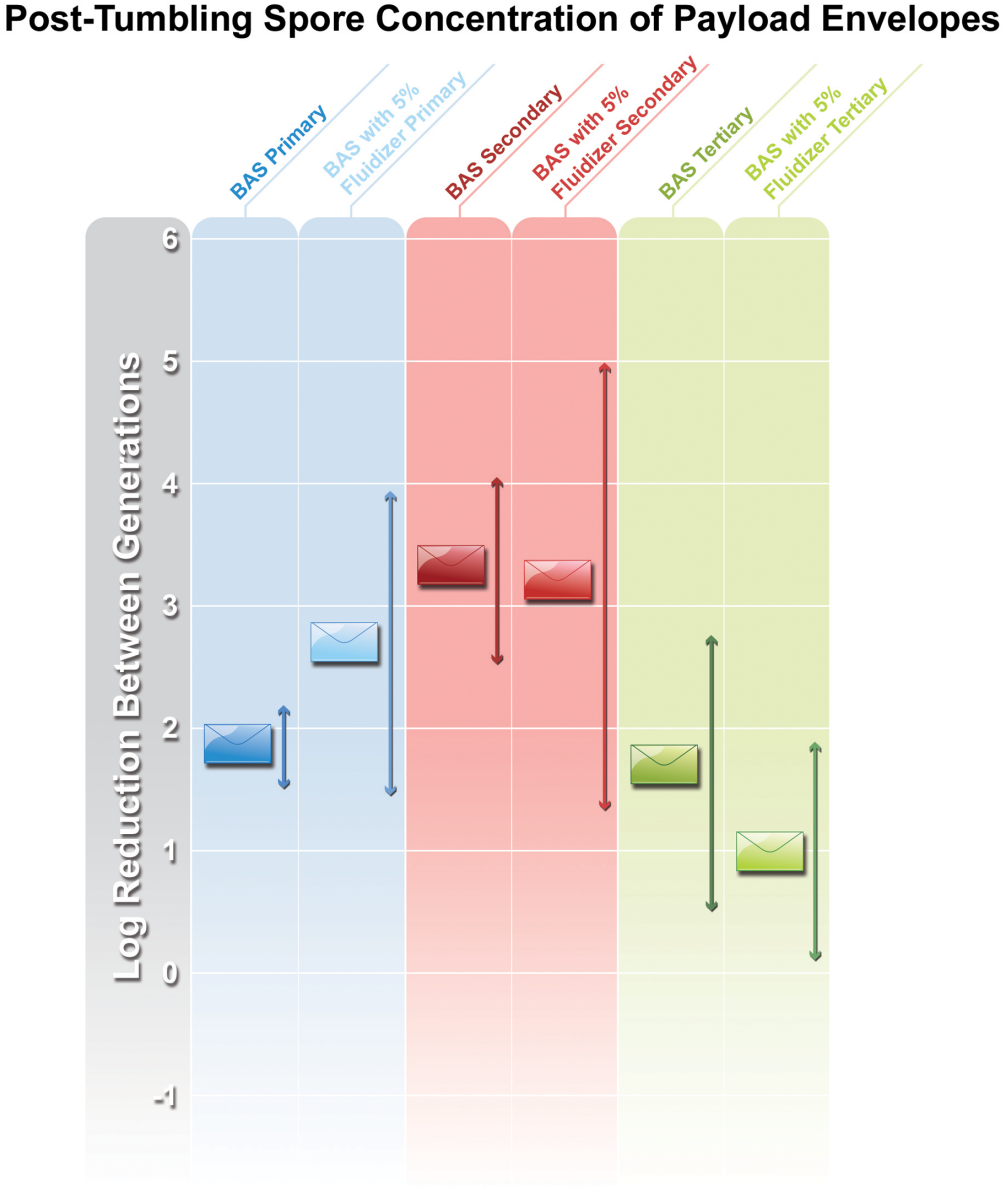
Log Reduction of spore concentration of payload envelopes. Arrows indicate coefficient of variation.

**Fig 3:**
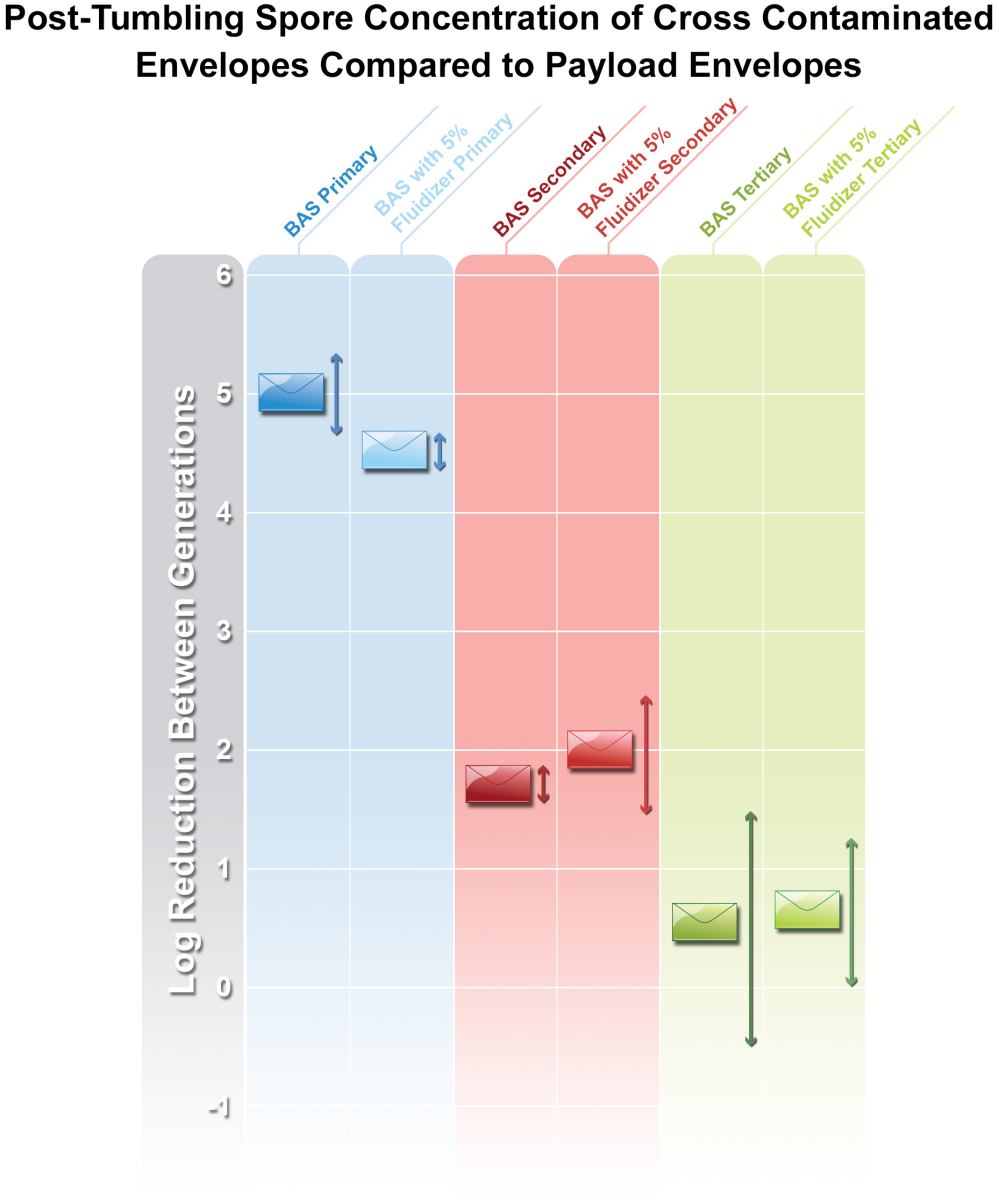
Post-tumbling spore concentration of cross-contaminated envelopes compared to payload envelopes. Arrows indicate coefficient of variation.

There was no statistically significant difference in the number of spores lost from the payload letters after tumbling nor was there a difference between preparations with respect to loss of spores from payload letters (Fig 2). However, there was a statistically significant difference and an observed pattern in the concentration of spores transferred from payload to cross-contaminated letters between generations (Fig 3). The addition of fluidizer is used in dry particle production to discourage agglomeration and make particles less likely to adhere to surfaces. However, in our study, there was no statistical significance in the number of spores recovered from envelopes exposed to both dry milled material and the spores prepared in the presence of fluidizer.

The chart in Fig 2 shows how the cross-contaminated mail can be compared to the load letter after tumbling in its respective generation. In the first generation of BAS tumbling, there was a roughly 5 log difference between the post-tumbled load letter with an initial 5 x 10^9^ CFUs g^-1^ loaded into the envelope, and the cross-contaminated mail. As noted, there was a significant amount of the spore powder preparation observed at the bottom of the bucket after the experiment was performed, suggesting that a substantial amount of material was able to escape the original payload envelope but did not adhere to the included envelopes for cross-contamination analysis. It is unclear whether or not the envelopes were saturated with spores or if more organisms could have bound to the surface, or if spores from the payload envelope did not come into contact with the additional letters in our experiment.

The third generation tumbling results showed less than half a log difference between the contamination source and the cross-contaminated envelopes in the third generation of BAS contaminated envelopes. These data have an impact on modeling and assessments of the degree of the spread of spores through the USPS and any other fomite transmission. The previous work performed with BG indicates that as the number of tumbling generations increase, the transfer of BG spores from contaminated mail to other envelopes will sharply decrease (15). The same experiment with BAS with and without 5% fluidizer, indicates that the potentially pathogenic material has physical characteristics which allow it to be more easily transferrable between fomites resulting in a higher number of contaminated pieces of mail but at diluted concentrations compared with the surrogate BG. The authors acknowledge that these results contradict the findings from the reaerosolization of spores using a finger to open sealed letters. Although the exact mechanism for the dislodging of the spores from the envelopes through tumbling or opening of letters was not investigated in this study, it is plausible that during the tumbling of the letters, static forces could have affected the transfer of spores from envelope to envelope in a way that is not observed with the mechanical process of opening a letter. The role of the extracellular proteins and surface structure of spores as it relates to adhesion to surfaces is an area of research deserving further investigation.

In the previously published manuscript on this topic, the authors point out that even after a series of three generations of tumbling, sufficient spores were present on cross-contaminated mail to generate an aerosol upon opening in excess of human LD_50_ estimates for inhalational exposure to *B*. *anthracis* spores [19]. The authors suggested that this reaerosolization of spores from letters opened or otherwise manipulated may provide a possible explanation for the fatal inhalation anthrax infections of the elderly woman in Connecticut and hospital supply worker in New York City, for whom no environmental positive samples were detected in their known surroundings [19]. The mechanical process of opening letters contaminated with BAS demonstrated that *Ba* spores may adhere to substrates differently than BG spores processed similarly. Despite the fact that the percentage of spores reaerosolized was nearly identical and the surface area to which they were adhered to were identical, the initial contamination of the BG coated letters were two logs greater than *Ba*. Repeating the previous experiments reported in Edmonds *et al*. 2010 using a non-pathogenic strain of *B*. *anthracis* gives validity to the idea that the concentration of spores present in those contaminated environments could have been below detection limits of environmental sampling technology used in the investigation of the Connecticut and New York City cases [8, 20–21].

There have been questions regarding the full extent of the spread of contamination during the 2001 Amerithrax event, which has in part been due to an incomplete understanding of how *Ba* spore contamination transferred during interactions between pieces of mail and with the environment, and the extent to which human exposures occurred resulting from these interactions. The two inhalation anthrax cases for which no positive environmental samples were discovered within the homes or workplaces of those individuals makes it conceivable, although speculative, that other individuals along the East Coast could have been exposed but not infected, or infected and not accurately diagnosed. However, enhanced public health case finding and surveillance activities did not identify any additional cases [6, 10], and rates and causes of death among workers at one of the USPS facilities impacted by the Amerithrax event during the year following the event were found to not differ significantly from rates for the previous 5 years [25]. However, it is important to include a broad scope of possible exposure locations and scenarios in a thorough evaluation and risk assessment of any bioterrorism event involving *Ba*, based on solid scientific understanding of the possible fate and transport of spores in such an event.
